# Late radiation toxicity in Hodgkin lymphoma patients: proton therapy's potential

**DOI:** 10.1120/jacmp.v16i5.5386

**Published:** 2015-09-08

**Authors:** Allison Toltz, Naomi Shin, Ellis Mitrou, Cecile Laude, Carolyn R. Freeman, Jan Seuntjens, William Parker, David Roberge

**Affiliations:** ^1^ Department of Physics McGill University Montreal QC; ^2^ Medical Physics Unit McGill University Health Centre Montreal QC; ^3^ Département de radio‐oncologie Centre Hospitalier de l'Université de Montréal Montreal QC; ^4^ Department of Radiation Oncology McGill University Health Centre Montreal QC Canada

**Keywords:** proton therapy, cardiotoxicity, secondary cancer, Hodgkin lymphoma

## Abstract

In 2010, all young patients treated for intrathoracic Hodgkin lymphoma (HL) at one of 10 radiotherapy centers in the province of Quebec received 3D conformal photon therapy. These patients may now be at risk for late effects of their treatment, notably secondary malignancies and cardiac toxicity. We hypothesized that more complex radiotherapy, including intensity‐modulated proton therapy (IMPT) and possibly IMRT (in the form of helical tomotherapy (HT)), could benefit these patients. With institutional review board approval at 10 institutions, all treatment plans for patients under the age of 30 treated for HL during a six‐month consecutive period of 2010 were retrieved. Twenty‐six patients were identified, and after excluding patients with extrathoracic radiation or treatment of recurrence, 20 patients were replanned for HT and IMPT. Neutron dose for IMPT plans was estimated from published measurements. The relative seriality model was used to predict excess risk of cardiac mortality. A modified linear quadratic model was used to predict the excess absolute risk for induction of lung cancer and, in female patients, breast cancer. Model parameters were derived from published data. Predicted risk for cardiac mortality was similar among the three treatment techniques (absolute excess risk of cardiac mortality was not reduced for HT or IMPT (p>0.05,p>0.05) as compared to 3D CRT). Predicted risks were increased for HT and reduced for IMPT for secondary lung cancer (p<0.001,p<0.001) and breast cancers (p<0.001,p<0.001) as compared to 3D CRT.

PACS numbers: 87.55.dh, 87.55.dk

## I. INTRODUCTION

Hodgkin lymphoma (HL) is the second most commonly diagnosed cancer among adolescents and young adults in the 12–29 age group.^(1)^ Treatment for early stage HL in Quebec in 2010 comprised two to four cycles of anthracycline‐containing chemotherapy followed by involved field radiotherapy (20–36 Gy). In such cases, five‐year overall survival is expected to approach approximately 95%.^(2)^ However, it is now well established that, in the longer term, these patients are at risk for thoracic radiotherapy treatment‐related death, in particular due to cardiovascular events and secondary malignancies.^3^, ^4^, ^5^ Cause‐specific mortality studies of patients with HL note secondary malignancy and cardiovascular failure as the second and third leading causes of death, respectively, after death due to HL.^(3)^ Following thoracic radiotherapy, one‐third or more young female patients will develop breast cancer.^5^, ^6^, ^7^, ^8^ Lung cancer is the second most commonly diagnosed secondary cancer among adolescent and young adult patients with HL, accounting for 13% of all secondary cancer diagnoses in this patient population.^7^, ^8^ Involved field radiotherapy (IFRT) at lower doses of 20‐30 Gy became the standard practice to treat supradiaphragmatic HL within the last 15 years, as compared to the traditional 40 Gy dose to a mantle field, resulting in insufficient follow‐up to fully document risk for late effects from lower (20–30 Gy) doses and smaller (involved field) treated volumes.^(9)^ It is anticipated that these lower doses and smaller fields will lead to a reduction in observed radiation‐induced cardiac mortality and secondary cancers. Although involved node radiotherapy (INRT) is considered by some to be the newest standard of care,^(10)^ this is not the case in our region and no patient was treated with INRT in the investigated period of this study.

Radiotherapy techniques and modalities providing greater normal tissue sparing may reduce the risks for cardiac mortality and secondary cancers.^4^, ^8^, ^11^, ^12^ Biological modeling using follow‐up information from patients receiving radiotherapy offers a method of interpreting the dose‐volume data of treatment plans to predict outcomes.^13^, ^14^ Dose planning studies have previously suggested that advanced radiotherapy techniques could reduce the dose to the organs at risk (OAR) considered in this work.^15^, ^16^, ^17^ Brodin et al.^(18)^ review models for risks of specific radiation‐induced effects in patients with HL, focusing on linear models that rely on mean dose for calculation of risk for cancer induction. Risk estimate comparison studies of IFRT techniques for HL, including volumetric‐modulated arc therapy and proton therapy by Maraldo et al.,^11^, ^19^ also use linear models for calculation of secondary cancer risk. In our work, the risk of radiation‐induced cardiac mortality was evaluated based on the dose‐volume distribution to the heart using the logistic relative seriality model, in accordance with the Quantitative Analysis of Normal Tissue Effects in the Clinic (QUANTEC).^(20)^ Risks for induction of secondary cancers in specific organs, lung and breast, were assessed using a linear quadratic model as modified by Schneider;^(14)^ this model adapts the concept of organ‐equivalent dose to account for nonhomogeneous dose distributions within an organ. Our study comprises a cohort assessment over a range of prescribed doses, which may mitigate the potential for bias in the evaluation of radiotherapy techniques with ideal or nonideal target volume location, as compared to a single‐patient case study. Helical tomotherapy (HT) and intensity‐modulated proton therapy (IMPT) plans were created using the original target volumes with unmodified planning goals, and the potential risks of cardiac mortality, lung cancer, and breast cancer associated with each modality were compared with the original clinically implemented 3D CRT plans.

## II. MATERIALS AND METHODS

With approval of the Research Ethics Board at 10 radiation oncology centers across the province, all treatment plans for patients under the age of 30 treated for HL during a six‐month consecutive period of 2010 were retrieved. Twenty‐six patients were identified and, after excluding patients with extrathoracic radiation or treatment of recurrence, 20 patients were replanned for HT and IMPT. Patient characteristics (gender, age, stage, chemotherapy regimen, subvolumes irradiated, and radiation dose) are given in Table 1

**Table 1:** Patient characteristics

*Patient Number*	*Gender*	*Age*	*Stage*	$Chemotherapy^{a}$	$Irradiated\;Volumes^{b}$	*Radiation Dose (Gy)*
1	F	28	IIA	none	2u, 3	20
2	M	17	IIIA	ABVE‐PC	1b, 2b, 3	21
3	F	15	IVA	ABVE‐PC	2u, 3	21
4	M	12	IVA	ABVE	1u, 2b, 3	21
5	M	21	IIBX	ABVD	1b, 2b, 3, 4u	21
6	F	14	IVA	ABVE‐PC	1b, 2b, 3	21
7	F	29	IIA	ABVD	2b, 3	21
8	M	17	IIA	ABVE‐PC	1u, 2b, 3	21
9	M	27	IIA	ABVD	2b, 3	21
10	M	23	IV‐EBX	ABVD	2u, 3	21
11	M	21	IIB	none	1u, 2b, 3	26.5
12	M	19	IB	ABVD	2u, 4u	30
13	F	28	IIIB	ABVD, ICE, BEAM	2u, 3	30
14	M	23	IIA	unknown	2b, 3	30
15	M	19	IIA	ABVD	2u, 3, 4u	30.6
16	M	26	IIB	ABVD	1b, 2b, 3, 4u	30.6
17	F	29	IIA	ABVD	2b, 3, 4u	30.6
18	F	20	IIAX	ABVD	1b, 2b, 3	30.6
19	F	26	IIA	ABVD	1b, 2b, 3	30.6
20	F	27	IIB	BEAM	1b, 2b, 3	36

a Chemotherapy regimen: ABVD=doxorubicin, bleomycin, vinblastine, dacarbazine; ABVE−PC=doxorubicin, bleomycin, vincristine, etoposide, prednisone, cyclophosphamide; ICE=ifosfamide, carboplatin, etoposide; BEAM = carmustine, etoposide, cytarabine, melphalan; none=no chemotherapy; unknown=unknown chemotherapy regimen administered.

b Key: 1=cervical,2=supraclavicular,3=mediastinum,4=axillary,u=unilateral,b=bilateral.

### A. Target and organ delineation

The planning target volume (PTV) as specified by the original treating physician was not modified for this study, and the new plans were referenced to the original PTV for the purposes of plan comparison. Organs at risk (OAR) for this work were specified as the heart, lungs, and, for female patients, breast tissues. Prior to replanning, these OARs were contoured for each patient in a consistent manner. Lung volume was identified using the Eclipse lung segmentation tool (v.8.9, Varian Medical Systems, Palo Alto, CA), heart volume was defined using the Feng heart atlas, and the breast volume was defined using the consensus definitions of the Radiation Therapy Oncology Group Breast Cancer Atlas.^21^, ^22^

### B. Treatment planning

Dose distributions resulting from original photon 3D CRT plans were imported into the treatment planning system in terms of absolute dose. All original plans comprised the same photon beam arrangement of parallel opposed anterior–posterior/posterior–anterior photon beams (photon energies: 6 MV, 10 MV, 18 MV, 23 MV) with multileaf collimation for field shaping. No modifications to the dose calculation parameters were made.

Treatment plans were generated for HT using TomoTherapy Hi·Art System (v.4.2, Accuray Inc., Sunnyvale, CA) with a 2.5 cm field width, 0.287 cm pitch, and a fine dose grid. The plans were optimized according to our institution's planning practices. Directional arm blocks were created to prevent the optimizer from allowing beams to enter the patient through the arms.

Proton treatment planning was performed using Eclipse treatment planning software (v.8.9) (Varian Medical Systems) with beam data of the Rinecker Proton Therapy Center, Munich, as provided by Varian. Planning adhered to recommendations of ICRU Report 78; notably, beamlines where a sensitive structure lay immediately distal to the target and beam angles of incidence that may have led to uncertainties in beam range due to possible misalignment were avoided.^(23)^ Plans were limited to four fields or fewer, in keeping with the clinical practices of proton therapy reference institutions.^(24)^ The modulated scanning proton beam mode with a range shifter allowing for nominal energies ranging from 68–250 MeV and the proton convolution superposition algorithm (v.8.08.9) were used for dose calculation. Proton plans were optimized to the original prescribed dose scaled by a factor of 1.1 to account for the relative biological effect.^(23)^

The planning goals for target coverage were specified at covering 100% of the PTV with 95% of the prescribed dose ($V_{95\%}=100\%$) without exceeding $V_{\max}=108\%$ for all three modalities in accordance with the goals of the original 3D CRT plans. The planning constraints for OARs for HT and IMPT plans were set at specific DVH points ($V_{Rx}$ for all structures, $V_{5\;\text{Gy}}$ and $V_{2\;\text{Gy}}$ for heart, $V_{20\;\text{Gy}}$, and $V_{5\;\text{Gy}}$ for lung, and $V_{2\;\text{Gy}}$ for breasts).

### C. Biological modeling

The differential dose‐volume histograms for HT and IMPT plans were exported and analyzed. The model for cardiac mortality was selected in accordance with the QUANTEC reviews on radiation dose‐volume effects in the heart.^(13)^ Excess risk of cardiac mortality at 15 years postirradiation was computed using the relative seriality model ((1), (2)), which is based on Poisson statistics to describe the probability of cell survival and incorporates the volume dependence of the radiation response of an organ.^(25)^ The probability of cell survival P(D) of a subvolume, i, irradiated to dose, D, is related to the 50% response dose $D_{50}$, the maximum value of the normalized dose‐response gradient for the endpoint γ and the seriality of the organ, s.^(25)^ The probability of toxicity in the organ, P, is the product of the probability of toxicity of each subvolume relative to its fractional volume Δv ((1), (2)). The parameter values used for this model are listed in Table 2, ^(26)^

(1) $$P(D)=2^{-e^{e\gamma \left(1-\frac{D}{D_{50}}\right)}}$$

(2) $$P={\left[1-\prod_{i=1}^{M}{\left[1-{\left[P(D_{i})\right]}^{s}\right]}^{\Delta v}\right]}^{\frac{1}{s}}$$

**Table 2:** Parameter values for models

*Parameter*	*Relative Seriality Model for Cardiac Mortality*	
$D_{50}$, dose at which 50% probability of complication (Gy)	70.3	
γ, maximum relative slope of the dose‐response curve for cardiac mortality	0.96	
*s*, relative seriality factor	1	
*Parameter*	*Modified Linear Quadratic (Schneider) Model for Breast Cancer*	*Modified Linear Quadratic (Schneider) Model for Lung Cancer*
$R_{f}$, repopulation and repair fraction	0.62	0.84
*α*, represents the repairable damage component from the linear quadratic model (${\text{Gy}}^{-1}$)	0.067	0.061
μ, slope of cancer induction from linear‐no‐threshold model (cases per 10,000 persons per year per Gy)	4.8	2.7

The Schneider modified linear‐quadratic model ((3), (4)) was used to predict the excess absolute risks (EAR) for induction of lung cancer and breast cancer.^27^, ^28^ Linear models are less sensitive to the inhomogeneity of a dose distribution in a nontarget organ as the dose‐volume information is condensed into a single mean dose parameter for the model calculation. The organ equivalent dose (OED) concept represents the risk of inducing carcinogenic mutations as a volume‐weighted sum of the risks to each subvolume upon irradiation. The dose‐volume data extracted from the treatment planning software are used to determine the dose to each subvolume of the organ. The number of mutated cells in a subvolume resulting in carcinogenesis, $M_{c}$, after one fraction of dose, D, is related to the original number of cells, $N_{0}$, and the tissue‐specific parameters representing the repairable damage component α and the repopulation and repair factor $R_{f}$.^(29)^ The risk to the whole organ is the product of the OED and the mutation parameter, μ, which represents slope of organ‐specific cancer induction with dose from the linear‐no‐threshold model as follows:

(3) $$M_{c}(D)=\mu N_{0}\cdot \frac{e^{-\alpha^{\prime }\cdot D}}{\alpha^{\prime }\cdot R_{f}}\left[1-2R_{f}+R_{f}^{2}e^{\alpha^{\prime }\cdot D}-{\left(1-R_{f}\right)}^{2}\cdot e^{\frac{\alpha^{\prime }\cdot R_{f}}{1-R_{f}}\cdot D}\right]$$

(4) $$EAR_{organ}=\frac{M_{c}(D)}{N_{0}}=\frac{1}{V_{organ}}\sum_{i}V_{i}\cdot M_{c}(D_{i})$$

Because induction of cancer due to radiation is a stochastic effect, the risk represents the population‐based probability for the induction of cancer for a specified number of person‐years of observation. The risk for an individual person in that population is the ratio of the number of excess cases per number of irradiated individuals in the population. The resulting EAR for secondary cancer is, therefore, expressed as the number of cases per 10,000 person‐years of observation. The parameter values used for this study are listed in Table 2, ^27^, ^28^ A paired *t*‐test was used to evaluate significance of cohort results.

## III. RESULTS

Risk results for each patient are listed in Table 3. The average risk ratio for cardiac mortality at 15 years postirradiation was *0.8* (p=0.4) for HT and *0.7* (p=0.1) for IMPT as compared to 3D CRT. The average risk ratio for lung cancer induction at 30 years postirradiation per 10,000 persons irradiated was 1.2 (p=0.001) for HT and 0.6 ($p=2\times 10^{-7}$) for IMPT as compared to 3D CRT. For breast cancer induction in female patients, this ratio per 10,000 persons irradiated was found to be 2.5 ($p=5\times 10^{-4}$) for HT and 0.4 ($p=4\times 10^{-4}$) for IMPT.

**Figure 1:**
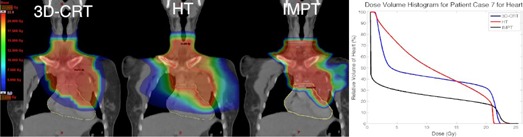
Patient case (Patient 7) demonstrating nonsignificant reduction in predicted risk of cardiac mortality for HT (middle) and IMPT (right) as compared with 3D CRT (left). PTV contour in red, heart contour in yellow. Relative DVH for heart for each modality shown on right.

**Figure 2:**
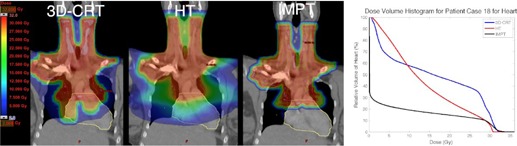
Patient case (Patient 18) demonstrating pronounced reduction of risk for cardiac mortality for HT and IMPT as compared with 3D CRT (left). PTV contour in red, heart contour in yellow. Relative DVH for heart for each modality shown on right.

Risk for cardiac mortality was not significantly reduced for the cohort for HT or IMPT relative to 3D CRT (p=0.4,0.1). A reduction in heart volume receiving low dose was observed for most patients for IMPT as compared to 3D CRT, as shown in Fig. 1, a patient case (Patient 7) representative of the cohort. However, this did not correlate with a significant reduction in risk for cardiac mortality as predicted by the relative seriality model (Table 3). This is likely due to the low probability for cardiac mortality in subvolumes receiving low doses.^(26)^ The most pronounced differences among the three modalities in risk of cardiac mortality were observed for Patients 18 and 20, both of whom had mediastinal involvement in the region anterior to the heart and lungs. Patient 20 was found to have the highest predicted risk of cardiac mortality with 3D CRT among the cohort and also received the highest prescribed dose. IMPT was predicted to reduce the volume of heart receiving the prescribed dose, $V_{\text{Rx}}$, to the heart from 20% to 1% and the excess risk of cardiac mortality at 15 years postirradiation from 7.7% to 1.7% for Patient 20. The reduction in risk for cardiac mortality is attributed to the reduction in heart volume receiving high dose. HT was also found to reduce the risk for cardiac mortality for Patient 20, but not as substantially as IMPT. A patient case (Patient 18) exhibiting reduction in risk for cardiac mortality for HT and IMPT relative to 3D CRT is shown in Fig. 2. The absolute risk results for the patient cohort are not in contradiction with other cardiac mortality risk estimates on an HL population.^11^, ^16^, ^30^ Thus, at the lower prescribed doses common to HL treatments (20–30.6 Gy), the excess risk of cardiac mortality at 15 years postirradiation was not found to be reduced for IMPT or HT as compared to 3D CRT.

**Table 3:** Results for excess risk of cardiac mortality at 15 years postirradiation and excess absolute risks for lung cancer and breast cancer at 30 years postirradiation for 3D CRT, HT, and IMPT. Dash (‐) indicates male patients for whom risk of breast cancer was not modeled

*Patient Number*	*Excess Risk of Cardiac Mortality (%)*			*Excess Absolute Risk for Lung Cancer*			*Excess Absolute Risk for Breast Cancer*		
				(cases per 10,000 person‐years observation)					
	3D CRT	HT	IMPT	3D CRT	HT	IMPT	3D CRT	HT	IMPT
1	0.1	0.1	0.1	8.2	11.4	5.1	3.9	13.2	2.1
2	0.0	0.0	0.0	7.3	9.9	4.2	–	–	–
3	0.0	0.0	0.0	7.1	10.1	5.6	1.5	4.3	0.8
4	0.0	0.0	0.0	9.9	12.0	9.4	–	–	–
5	0.0	0.0	0.0	9.5	12.8	9.0	–	–	–
6	0.1	0.0	0.0	10.7	12.3	8.8	11.0	17.5	6.6
7	0.1	0.0	0.0	10.5	12.0	4.3	6.9	13.6	2.7
8	0.1	0.0	0.0	8.3	11.3	4.1	–	–	–
9	0.1	0.1	0.1	9.2	12.6	5.3	–	–	–
10	0.1	0.1	0.1	8.5	11.8	5.4	–	–	–
11	0.2	0.1	0.1	11.7	13.8	6.7	–	–	–
12	0.0	0.0	0.0	3.7	6.6	1.7	–	–	–
13	0.0	0.0	0.0	6.4	9.5	3.5	1.0	3.4	0.0
14	0.8	1.8	0.6	9.5	11.9	7.9	–	–	–
15	0.1	0.0	0.0	8.7	1.0	3.2	–	–	–
16	0.6	0.4	0.4	11.0	11.7	7.9	–	–	–
17	1.1	1.0	1.1	13.4	16.2	8.4	5.9	9.2	2.6
18	1.3	0.6	0.5	14.5	16.9	8.2	4.0	10.8	1.1
19	1.5	1.4	1.5	13.0	15.6	6.0	3.3	7.1	0.5
20	7.4	2.1	1.1	14.0	17.5	6.2	7.1	19.0	2.0

**Figure 3:**
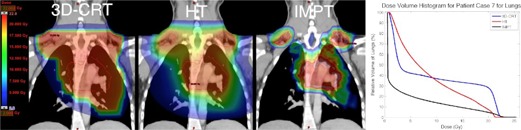
Patient case (Patient 7) exhibiting reduction in risk for lung cancer for IMPT and increase in risk for lung cancer for HT as compared to 3D CRT. PTV contour in red. Relative DVH for lungs for each modality shown on right.

Risk for induction of lung cancer was predicted to be reduced for all patients for IMPT as compared to 3D CRT and to be increased for all patients for HT as compared to 3D CRT. A patient case (Patient 7) representative of the cohort is shown in Fig. 3; the HT lung DVH illustrates the decrease in volume receiving high dose at the expense of the volume receiving low dose, while IMPT reduces lung volume receiving both low and high dose.

**Figure 4:**
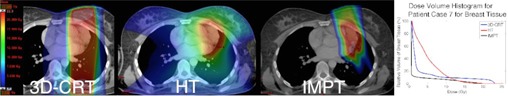
Female patient case (Patient 7) exhibiting reduction in risk for breast cancer for IMPT and increase in risk for breast cancer for HT as compared to 3D CRT. PTV in red. Relative DVH for breasts for each modality shown on right.

Risk for induction of breast cancer in female patients was predicted to be reduced for all female patients (9 of 9) for IMPT as compared to 3D CRT and to be increased for all patients for HT as compared to 3D CRT. A patient case (Patient 7) representative of the female patients in the cohort is shown in Fig. 4. HT was observed to reduce the volume of breast tissue receiving high dose at the expense of the volume of breast tissue receiving low dose, while IMPT plans reduced the volume of breast tissues for all doses. This is most notable in the contralateral breast, which is completely spared for IMPT, while the volume receiving low dose is largely increased for HT. Our cohort results are in agreement with the study by Maraldo et al.^(11)^ where a linear model was used to predict risk for breast cancer induction and with Cella et al.^(30)^ where a single patient case was considered. Breast sparing techniques were not implemented in our study as they were not considered in the original treatment aims; however, the implementation of such could be used to further investigate the EAR for breast cancer posed by HT techniques.

## IV. DISCUSSION

IMPT was hypothesized to reduce late normal tissue toxicity due to better normal tissue sparing achievable with this modality as compared to 3D CRT and HT in the treatment of HL. This potential advantage was investigated in this work using biological models to predict the risks for specified late effects in order to compare these modern radiotherapy techniques on a large‐scale patient cohort comprising all patients treated with involved field radiotherapy for HL in the province of Quebec within a six‐month period of 2010. The aim was to establish the extent of the potential risks of late effects of 2010 radiotherapy techniques on a regional patient population and to compare these to the potential risks associated with radiotherapy delivered using HT and IMPT in the context of 3D CRT.

A number of assumptions were made in this work, which may contribute to uncertainty in the results. Applying the PTV concept as described in ICRU Report 78 may not be appropriate for proton therapy due to differences in uncertainties that are specific to proton therapy delivery.^(23)^ Proton beam dose distribution is subject to 3.5% uncertainty in beam range due to uncertainty in conversion of CT Hounsfield unit to proton stopping power and multiple coulomb scatter processes.^(31)^ This implies that lateral margins to the CTV are generally different from margins required in the beam axis direction. These differing uncertainty margins were not taken into consideration in this work as it was a planning comparison study necessitating equivalent target definitions and goals. A target volume with larger distal margins presents an increased risk to OARs in this region, though current practice mitigates this through prudent selection of beam direction by an experienced planner.

Inconsistencies were observed as a result of the nature of a multi‐institutional retrospective study. Procedures for planning including CT simulation acquisition parameters, use of heterogeneity corrections, plan normalization, and OAR delineation varied from one center to another. These differences were considered negligible (in the case of CT acquisition parameters and heterogeneity corrections) or corrected for (in the case of plan normalization and OAR delineation). Our aim was to predict the risk of late effects of differing modalities of radiotherapy based on the actual treatment plans. Though repositioning with arms above the head would be better suited to both HT and IMPT, patient positioning was limited to the position at 3D CRT simulation scanning. The OARs in this work are partially out of the treatment field for 3D CRT plans, and Eclipse TPS is known to underestimate dose outside the field.^(32)^ However for partially in field organs receiving >5% of the prescribed doses (the heart and lungs in this study), the TPS DVH can be used.^(33)^ A conservative estimate of the effect of the variation of these treatment planning parameters, which together might result in a variation in the absolute dose of no more than 10%, would result in an uncertainty of the risk results of 0.5% for excess risk of cardiac mortality and 10% for EAR for secondary cancers. Predicted risk for breast cancer for 3D CRT and HT plans may be a conservative estimate, as the majority of the organ receives <5% of the prescribed dose and dose to this region is underestimated by TPS.^(32)^ If accounted for this would only increase the disparity between risk from IMPT as compared to photon techniques. This would not alter our overall conclusions as determined by the ratio of risks for each modality rather than absolute values.

In the comparison of IMPT to photon 3D CRT, the favorable dose distribution exhibited by proton beams may be compromised by secondary particle contamination. This was deemed negligible for this work considering that the secondary neutron dose during spot scanned delivery of a proton beam has been modeled and measured.^34^, ^35^ The effective neutron dose outside the target for actively scanned protons was measured to be on the order of mSv/Gy delivered for a prostate treatment case.^(35)^ Neutron dose is affected by heterogeneity in the path of the primary proton beam, the incident proton beam energy, and energy and position of neutrons generated, all of which are factors determined by the anatomical aspects of a proton treatment. This complicates efforts to make simple or analytical estimates and necessitates measurements or simulations of clinical application for accurate quantification.

In contrast to passively scattered proton delivery where the dominant source of externally generated neutrons is the field‐shaping devices, the absence of field‐defining apertures and range compensators in magnetically steered pencil beam scanning delivery with intensity modulation results in significantly reduced neutron production.^(36)^ Thus, even the most conservative estimation of the relative biological effect (RBE=20) of neutron dose would still result in a neutron dose on the order of tens of milliSieverts per treatment Gray, and its inclusion in modeling risk estimates would not increase risk results for cardiac mortality or EAR for secondary lung or breast cancers. This conclusion is in agreement with similar studies.^36^, ^37^, ^38^, ^39^ This work represents a first estimation of risk due to different modalities as planned by commercially available TPS, which may be further refined in future work with Monte Carlo dose calculation to take RBE into account.

Oversimplifications of biological and physical responses to radiation, inherent to the modeling process, add a further degree of uncertainty to the results. There are known confounding factors in the development of cardiac mortality that were not accounted for in this work such as smoking, hypertension, obesity, and family history, all of which may play an important role with regard to heart disease. Chemotherapy affects both the risks of cardiotoxicity and developing secondary malignancies, while genetic susceptibility and age at irradiation may also affect the risks of developing secondary malignancies.^3^, ^5^, ^13^ Radiation damage to the heart has been found to affect radiation‐induced lung damage.^(40)^ While it may also be argued that comparing metrics of death due to cardiotoxicity to induction of secondary cancers is unequal, predicting induction of cardiac morbidity is difficult due to the high rate of occurrence in asymptomatic patients, both irradiated and nonirradiated.^(13)^

**Figure 5:**
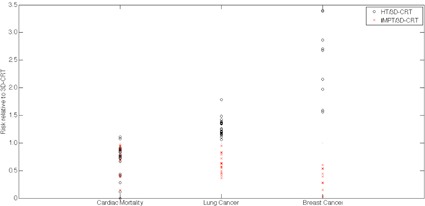
Risk for cardiac mortality, lung cancer, and breast cancer for HT (black circles) and IMPT (red crosses) relative to 3D CRT for all patients in the study.

Ultimately, numerous uncertainties are present in this work as a consequence of the nature of the study. A radiobiological model is limited by the goodness of fit and the variability of the data. The multimodal, multi‐institutional radiotherapy comparison study is subject to inconsistency in structure definition, imaging acquisition, patient positioning, and institutional or physician specific practices. Treatment planning algorithms are less accurate than Monte Carlo simulations for calculating dose distributions and inconsistent with respect to inclusion of out‐of‐field and secondary particle contributions. Some uncertainties, such as patient positioning, have a more pronounced effect for proton therapy as compared to photon therapy. Nonetheless, comparison studies have been found to have greatly minimized these uncertainties when the risks for proton techniques are compared in relation to the photon techniques, as opposed to comparing the absolute risk values.^41^, ^42^ The conclusions of this study are, therefore, based on the comparison of the treatment modalities by the ratio of risks for HT and IMPT as compared to 3D CRT to provide a more meaningful method for evaluation. Figure 5 illustrates the variability over the patient cohort for risk of each late effect considered relative to 3D CRT for HT (black circles) and IMPT (red crosses), where relative risk (RR) values below 1 represent a decrease in predicted risk for a given patient and treatment modality and RR values greater than 1 represent an increase in predicted risk.

The focus of this work was to investigate the risk of late, life‐threatening effects of radiotherapy treatments for HL. For this reason, examination of other biological endpoints, such as radiation pneumonitis, spinal cord injury, esophageal stricture and esophagitis, hypothyroidism, and cardiac perfusion defects, were considered to be outside the scope of this work.^43^, ^44^, ^45^, ^46^, ^47^, ^48^ Similar planning comparison studies for HL include those by Maraldo et al.^(19)^ in which patients with HL, including adults, were replanned from INRT with 3D CRT to volume‐modulated arc therapy (VMAT) and unspecified application of proton therapy. Their work derived a linear dose model to evaluate excess risk of stroke using mean dose to carotid arteries, while our study examines risks to whole heart for damage resulting in cardiac mortality with HT and IMPT and uses a model that reflects the observed dose‐volume relationship in the heart. Another study by Maraldo et al.^(11)^ examined risks for cardiac mortality to patients treated for HL using a logistic dose‐response model, as well as radiation induced lung and breast cancers using linear models using for INRT comparing 3D CRT with VMAT and IMPT. Our study uses the relative seriality model, as presented in QUANTEC reviews, to evaluate risk for cardiac mortality and a modified linear quadratic model for evaluation of secondary cancers.^(13)^ Brodin et al.^(18)^ presented a tool for estimation of acute and late effects in patients with HL treated with radiotherapy. However, neither the relative seriality model for cardiac mortality nor the modified linear quadratic models were examined. In using these more detailed models to predict probability of mortality in subvolumes and probability of carcinogenic mutation in subvolumes, our work represents a rigorous risk modeling and treatment planning study on this important patient population. Cella et al.^(30)^ used the modified linear quadratic model, or organ‐equivalent dose model, to compare 3D CRT to HT and proton therapy for HL, but only for a single patient case. In replanning a patient cohort, we have attempted to mitigate any inherent bias towards particular cases that may be well‐suited or poorly‐suited to proton therapy.

## V. CONCLUSIONS

Twenty patients under the age of 30 who received 3D conformal thoracic radiotherapy for HL at one of 10 institutions in 2010 were replanned for HT and IMPT. Risk of cardiac mortality was modeled using the relative seriality model and was not predicted to be significantly reduced (p=0.4,0.1) using HT photon techniques or IMPT as compared to 3D CRT for adolescent and young adult patients with mediastinal HL. In specific cases where target volumes were located anterior to the heart, a lower risk of cardiac mortality was predicted for both HT and IMPT as compared to 3D CRT. Using a modified linear quadratic model to predict risk of radiation‐induced cancers, IMPT was predicted to result in lower risks of induction of lung cancer and breast cancer for female patients when compared to the use of photon techniques. Notably, HT was predicted to yield increased risks of induction of these cancers as compared to 3D CRT. IMPT, as compared to 3D CRT or HT, is thus predicted to decrease the risks of radiation induced cardiac mortality for certain cases, and to reduce the risks for secondary lung and breast cancers for young patients receiving thoracic radiotherapy for HL.

## ACKNOWLEDGMENTS

Ms. Toltz reports funds from Fonds de Recherche en Santé du Québec (FRSQ) during the conduct of the study, and funds from the Natural Sciences and Engineering Research Council of Canada (NSERC) Collaborative Research and Training Experience (CREATE) program outside the submitted work. Dr. Laude reports grants from Fondation de France and from TomoTherapy during the conduct of the study, personal fees from Centre Leon Berard, Lyon, France, and grants from Elekta outside the submitted work. Ms. Shin, Dr. Roberge, Dr. Freeman, and Mr. Parker report grants from FRSQ during the conduct of the study. Dr. Seuntjens reports grants from FRSQ during the conduct of the study and grants from NSERC and from the Canadian Institutes of Health Research (CIHR) outside the submitted work.

## Supporting information

Supplementary Material

Supplementary Material

Supplementary Material

Supplementary Material
