# Supplementary figures and images for: Late radiation toxicity in Hodgkin lymphoma patients: proton therapy's potential
Source: J Appl Clin Med Phys. 2015 Sep 8;16(5):167–78. doi: 10.1120/jacmp.v16i5.5386 (PMC5690189; doi:10.1120/jacmp.v16i5.5386)

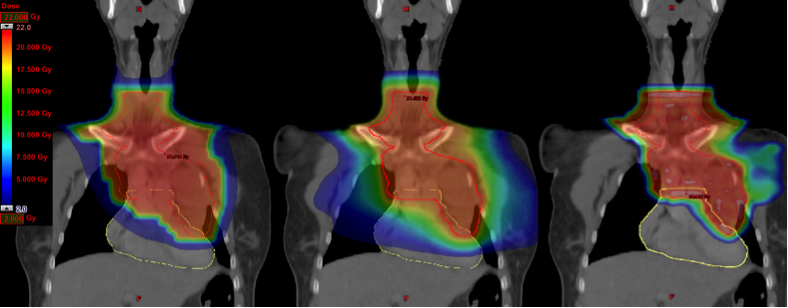

Supplement: Supplementary file 1 — Supplementary Material [file ACM2-16-167-s001.png]

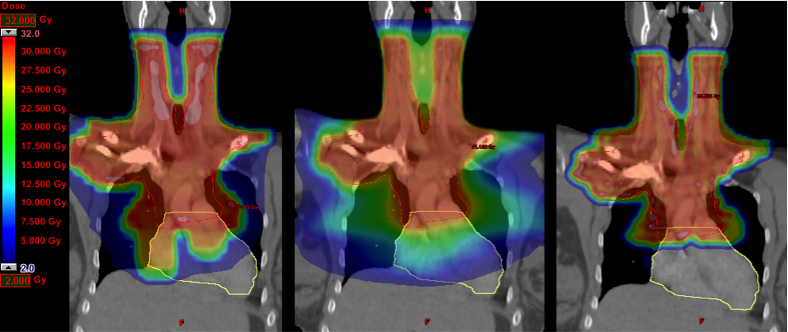

Supplement: Supplementary file 2 — Supplementary Material [file ACM2-16-167-s002.png]

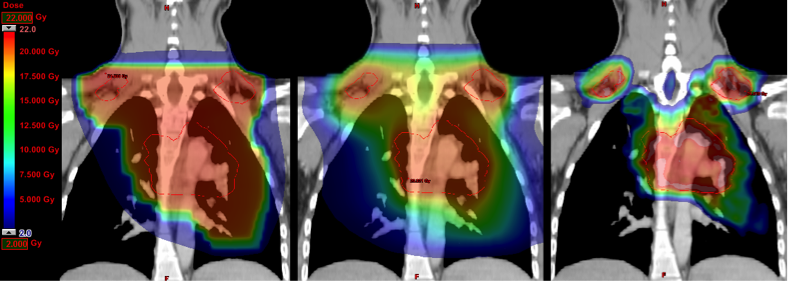

Supplement: Supplementary file 3 — Supplementary Material [file ACM2-16-167-s003.png]

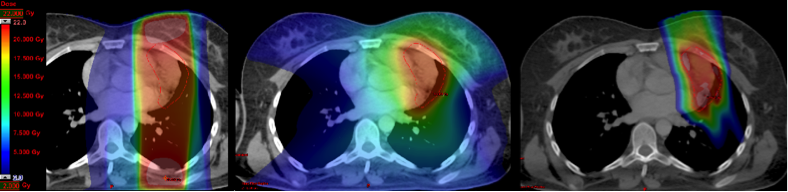

Supplement: Supplementary file 4 — Supplementary Material [file ACM2-16-167-s004.png]
